# Acupuncture mechanism studies employing task-based fMRI: a scoping review protocol

**DOI:** 10.1186/s13643-022-02007-1

**Published:** 2022-06-22

**Authors:** Yan Yan, Ru-ya Sheng, Yu Wang, Chun-hong Zhang

**Affiliations:** 1 Department of Acupuncture, The Third Affiliated Hospital of Zhejiang Chinese Medical University, Hangzhou, 310007 Zhejiang China; 2 Department of Acupuncture, The First Teaching Hospital of Tianjin University of Traditional Chinese Medicine, Tianjin, 300193 China; 3 Department of Acupuncture, Integrated Traditional Chinese and Western Medicine, The Third People’s Hospital, Changzhou, 213001 Jiangsu China

**Keywords:** Acupuncture, fMRI, Mechanism study, Scoping review

## Abstract

**Background:**

Acupuncture is a widely used alternative and complementary therapy. Functional magnetic resonance imaging (fMRI) is an important technique to explore the underlying mechanism of acupuncture, and the task-based fMRI can reflect the instant effects or sustained effects of acupuncture in the brain. This scoping review aims to summarize the characteristics of acupuncture mechanism studies employing task-based fMRI and conclude a reference for future studies.

**Methods/design:**

This review will follow the Guidance for Conducting Scoping Reviews. Eligible articles will be collected from 7 databases (PubMed, Embase, Cochrane, CNKI, WanFang, VIP, and CBM) with the related keywords such as “Acupuncture” and “fMRI”; those articles should be published from January 1, 2000, to December 31, 2021; and the language should be restricted in English or Chinese. Each research step will involve at least two reviewers. The PRISMA-ScR (Preferred Reporting Items for Systemic Reviews and Meta-Analysis Extension for Scoping Reviews) will be used to organize the review. Data will be extracted from the illegible articles, and findings will be presented in tables and narrative form. A descriptive qualitative approach to analysis will be conducted to form the scoping review.

**Discussion:**

This review aims to clarify the extent of acupuncture mechanism studies employing task-based fMRI. It is supposed to make a critical evaluation or propose quality requirements for future studies by summarizing the objectives and designs of eligible studies. What is more, directional suggestions will be provided for further studies.

**Scoping review registration:**

Open Science Framework https://osf.io/zjrdc/.

## Background

### Rationale

Acupuncture, as an indispensable component of traditional Chinese medicine (TCM), has been rooted and prospering in China for more than 2000 years [1] and has gradually been accepted as an alternative and complementary non-pharmaceutical therapy worldwide. Meanwhile, an increasing number of research have been conducted to explore the underlying mechanism of its clinical effects. The acupuncturist inserts needles into some different and specific locations on the body, which are usually called acupoints, to treat specific diseases [2]. Traditionally, Chinese acupuncture practitioners are accustomed to operating acupuncture following the theory of acupoint and meridian based on TCM [3]; nowadays, the modern natural science theory and methodology are supposed to be applied to reveal the underlying mechanism of acupuncture effect.

Since the last century, a large number of studies of acupuncture have been conducted with various objectives from different aspects globally. On the one hand, some studies are carried out among healthy volunteers to explore the substance of meridian, acupoint, or acupuncture effect [4, 5], on the other hand, some are conducted among patients in a pathological state. All these studies demonstrate the mechanism of acupuncture from different angles. During the treatment, both the practitioner and subjects can have the feeling of “Deqi” which refers to the needle sensation [6, 7]. Recent studies have shown that the central nervous system (CNS) plays a primary role in the acupuncture effect [8–10]. Similar to other studies on brain function, neuroimaging technologies have been extensively employed in this area, such as functional magnetic resonance imaging (fMRI) [11, 12], positron emission tomography (PET) [13], electroencephalography (EEG) [14], and magnetoencephalography (MEG) [15]. A study has shown that fMRI and PET/PET-CT are the two most commonly used methods [16]. Compared with PET-CT, fMRI has the advantages of being non-invasive, non-radiative, and no contrast agent. fMRI explores the functional activities and network connectivity of the brain by acquiring the change of blood-oxygen-level-dependent (BOLD) signal in different experimental conditions [17, 18]. BOLD shows the immediate oxygen consumption and the change of blood flow related to the neural activities of regional brain tissues which can laterally reflect the actual neural electrical activities.

### Definitions and concepts

fMRI includes two main types and they are resting-state fMRI (rs-fMRI) and task-based fMRI (tb-fMRI). Rs-fMRI or task-free fMRI requires the subjects to be in a “default” or “idle” state as much as possible [19], while tb-fMRI collects signal changes triggered by the predesigned tasks or a passive stimulus [20]. Acupuncture is an input-based passive stimulus, and current studies of its underlying effect mechanism can also be divided into these two types.

The rs-fMRI studies of acupuncture normally aim to observe the changes of BOLD before and after a course of acupuncture treatment [21–23]. Subjects in this kind of study are always patients but not healthy volunteers. Multiple acupoints that possess specific clinical effects are involved. The course of treatment is usually longer than 4 weeks, during which administrations are performed several times a week. These kinds of studies are similar to normal case-control studies or randomized controlled trials, which have pre-set primary outcomes. The data of fMRI in these studies are acquired as a secondary outcome before and after the intervention, and the main objective is to assist other outcomes to evaluate the clinical effect of the acupuncture intervention, or to explore the mechanism of acupuncture effect in CNS. The subjects in these studies should be in a silent state with restricted hearing and sight and no external stimulus should be given. Then, many different analysis methods of the fMRI data, such as Regional Homogeneity (ReHo) [24], amplitude of low-frequency fluctuation (ALFF) [25], functional connectivity (FC) [21], and independent component analysis (ICA) [26], can be processed to estimate the activations caused by the acupuncture stimulation. Generally, rs-fMRI is used to explore the intrinsic functions’ segregation or specialization of brain networks [27], while the acupuncture fMRI studies can make contributions to the research of brain function.

One of the focuses of this research is tb-fMRI employed to explore the changes in brain activation during the acupuncture stimulation or a period after stimulation [28–33]. These studies last for shorter time-taking healthy volunteers or patients as subjects. Besides, they can also be controlled studies involving both healthy volunteers and patients. One or two acupoints are selected in these studies, and a one-time acupuncture administration is given in one experimental program. These studies usually aim to discuss the instant effects [31] and sustained effects [32] of acupuncture. According to the theory of TCM, acupuncture may induce long-lasting post-administration effects [33]. Therefore, studies that discuss the brain changes causally related to the tasks of acupuncture stimulation should be classified as tb-fMRI. Such studies require a series of pre-set acupuncture events or tasks to induce exact activations in the brain. Meanwhile, tb-fMRI studies are more pertinent to explore more detailed underlying mechanisms of special manipulations, specific acupoints, and special needle materials. In terms of the program design, it varies because of the various research objectives and researchers’ different understandings of fMRI.

The most common design of tb-fMRI is the block-design task [28], also called the “on-off” model design, which requires a stimulus (on-state) followed by a resting state (off-state) appearing alternately several times during the scan to locate the activations. In the operation, the fMRI data are collected after acupuncture needles have penetrated the skin. Then the signal of activations is acquired during on-state with a continuous needle manipulation while the baseline signal is collected during off-state with no more manipulation. In the later stage, single-block design, also called modified block-design, has been developed to achieve different objectives.

As above mentioned, there exist sustained effects after acupuncture is given. Therefore, the signal collected in a block design under the off-state will not ideally return to the baseline. A new paradigm called not-repeated event-related (NRER) design has been developed to adapt to diverse objectives [33]. Such a design requires a specific “event,” which refers to an independent acupuncture manipulation without the acquisition of fMRI data which should be conducted before and after the “event” with the subjects in a silent state. The changes of brain activity are directly induced by the acupuncture event, and those changes reflect the sustained effects of acupuncture. Hence, the NRER is classified as tb-fMRI. The aforementioned data analysis methods, ReHo [34], ALFF [35], FC [36], and ICA [37], can be introduced to diversify the later data processing. What’s more, the paradigm of repeated measure NRER has also been designed to investigate the changes of the declining effects after the acupuncture administration [30].

At present, an abundant amount of acupuncture effect underlying mechanism studies employing tb-fMRI have been carried out with various objectives. Some of them only focus on exploring the brain activations of specific manipulations or specific acupoints to provide references for further research [38]; some of them discuss the differences between the true acupuncture and sham acupuncture, true acupoints and non-acupoints, in other words, the placebo effect of acupuncture [39]; some of them investigate whether acupuncture has clinical effects for diseases, or the effects in CNS [40]; some of them explore the specificity of acupoints, which means the different brain activities caused by manipulation in different acupoints [41]. Besides, there are some other special objectives. All studies of complex mixed design combining the acupuncture events with active movements or passive stimulations are also conducted for complex purposes [42–44].

Although fMRI has been widely employed in acupuncture mechanism studies, there is no standard for the task design of acupuncture. The factors that influence the acupuncture effects are complicated, such as the choosing of needle types, the manipulations of manual acupuncture, and the differences in acupoint selection. It is also exceedingly difficult to statistically analyze many small-sample studies following different design standards. Therefore, future studies require rigorous research designs to highlight the objectives so that the fMRI data can be acquired more efficiently. Hence, it is necessary to make a review of existing studies and a statistical description of the factors related to the experiments is needed.

### Objective

As applied techniques, both acupuncture and fMRI possess strong professionalism. The combination of these two techniques aims to reveal the deep mechanism of the acupuncture effect. The purpose of this scoping review is to identify, examine and summarize the status of the design of acupuncture mechanism study employing task-based fMRI.

## Method/design

This scoping review protocol was finished following the relevant sections of the Preferred Reporting Items for Systematic Reviews and Meta-Analyses extension for Scoping Reviews (PRISMA-ScR) guideline [45]. The final version of this review will be completed under the principle of the Guidance for Conducting Scoping Reviews [46].

When science is emerging, a scoping review is of great importance. It allows for a broad view of the nature and range of evidence of a given topic [47]. Besides, it offers a framework that would allow for greater breadth to include all pertinent acupuncture research employing tb-fMRI, irrespective of quality. What’s more, this approach can be applied to examine and identify the gaps in the evidence.

### Research questions

The purpose of this scoping review is to identify, examine and summarize the status of the design of acupuncture mechanism study employing task-based fMRI. The research questions guiding this review are:

1. What are the research scope and characteristics of the published literature on acupuncture employing task-based fMRI?
2. How can we conduct an acupuncture task-based fMRI study?

### Inclusion criteria

#### Participants

This review will include the clinical studies conducted on human beings. Subjects can be healthy volunteers or patients in a pathological state, and their disease, gender, age, handedness, allocation of the groups, dropout, and other basic information will be extracted as results. Besides, the pathological state of subjects in one study should be accordant with those in the other.

#### Concept

The studies discussed in this review should be those that take acupuncture as the only or the main intervention, employ tb-fMRI as an outcome measurement, and are completed with detailed descriptions.

Traditional acupuncture intervention types (manual acupuncture, electro-acupuncture, scalp needle, auricular acupuncture) will be considered while the moxibustion and unconventional acupuncture interventions (laser acupuncture, dry acupuncture, catgut embedding acupuncture, intradermal needle, or floating needle) will be excluded.

Task-based fMRI will be the focus of the study, in which acupuncture performance is designed in the task to explore its instant or sustained effects. Besides, the studies that only apply rest-state fMRI will be excluded. Most literature does not clearly explain the paradigms of design, so a full-text review of each fMRI-employed study is needed to identify whether it will be included. For the case-controlled trials conducted in a pathological state, an outcome measurement for therapeutic evaluation is necessary to demonstrate the effect of acupuncture, and it should be connected with fMRI results.

#### Context

This review will explore the eligible clinical studies included in 7 databases of PubMed, Embase, Cochrane, Chinese National Knowledge Infrastructure (CNKI), WanFang database, VIP database, and Chinese Biomedical Medicine disc (CBM). The year range of publication shall be from January 1, 2000, to December 31, 2021. The language shall be Chinese or English.

#### Study types

The review will include the experimental or quasi-experimental study of randomized controlled trials, non-randomized controlled trials, cross-over design trials, and case series studies. However, the small sample case reports that mainly focus on clinical effects will be excluded.

### Search strategy

The initial search was finished by having a quick browse of topics of interest on PubMed and it was confirmed that there were no similar studies. Besides, the keywords needed in the search were determined.

The systematized search will be conducted in the 7 databases of 3 in English language and 4 in Chinese language, PubMed, Embase, Cochrane, CNKI, WanFang, VIP, and CBM. Articles in other languages will be excluded. The year range will be set from January 1, 2000, to December 31, 2021. The whole searching strategy of PubMed is demonstrated in Table 1, and the searching field will be limited to “title/abstract.”

**Table 1:** Search strategy used in PubMed database

No.	Search items
1	fMRI
2	Functional MRI
3	Functional magnetic resonance imaging
4	OR/1-3
5	Acupuncture
6	Acupuncture therapy
7	Electroacupuncture
8	Acupoint
9	OR/5-8
10	4 AND 9

The reference list of all the identified articles will be searched again for additional studies. Reviews, diploma dissertations, conference articles, animal studies, and protocols for studies will not be involved.

### Study selection

**Fig. 1:**
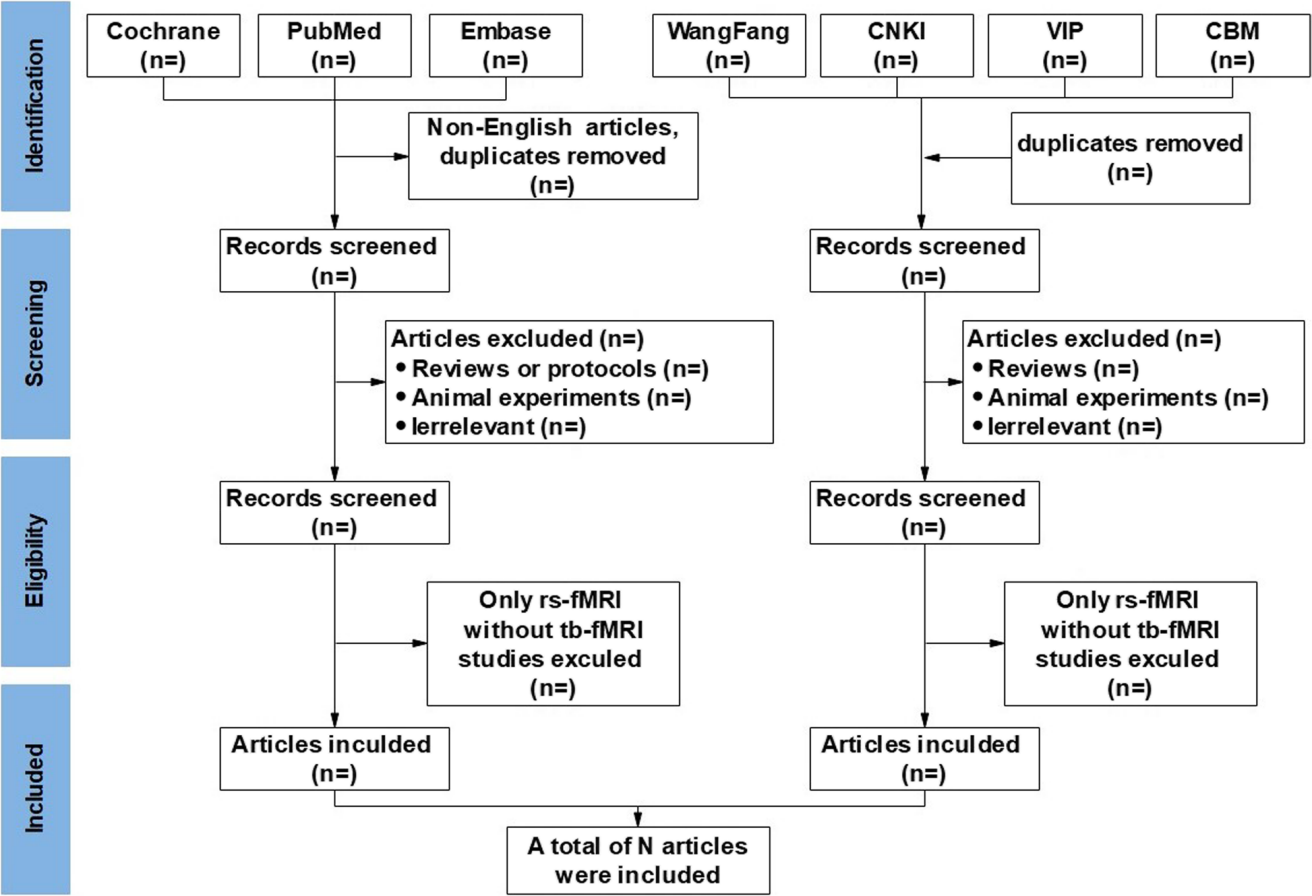
Process and results of the study selection

Following the search, all identified studies will be uploaded to Endnote X9.2 and duplicates will be removed. The non-English language articles from PubMed will also be removed. Titles and abstracts will be screened by two independent reviewers (YY and SRY) guided by the inclusion criteria. If a study cannot be identified whether it is eligible or not from titles and abstracts, the full text will be browsed. Then, the 2 reviewers will conduct a full-text review of the selected studies to further exclude the ineligible studies. Next, those literatures will be cross-checked and problems will be solved by the 2 reviewers. If there are any disagreements, the decision will be made by a third reviewer (CHZ). The process and results of the study selection will be presented in Fig. 1.

### Data extraction

Data will be extracted from eligible articles using Excel 2019. The data extraction table will be developed based on the data items detailed below. Any disagreements between the 2 reviewers will be solved through discussion or by referring to a third reviewer. Unreported data will be obtained by contacting the author through the mail. Modifications will be detailed in the final scoping review.

### Data items

The following data items will be collected during the data extracting process:

1. Publication characteristics (title, author, year of publication, country of study, the language of the report).
2. Study design (objective of the study, controlled method or not, numbers of groups, sample size, dropout)
3. Characteristics of participants (state or disease of participants, age, gender, handedness)
4. The paradigm of acupuncture task (block-design, signal block, modified block, event-related, NRER, repeated measure NRER, mix design)
5. Details of acupuncture intervention (acupoints, needle stimulation, response sought, needle retention time or not, numbers of experiment and their interval, texture of needle, other components of the intervention, controlled intervention, practitioner)
6. Details of fMRI acquisition (brand of fMRI scanner, magnetic field intensity, TR and TE of the scan, the state of subjects under scanning, numbers of processes, duration of each process)
7. Details of data preprocessing (standard template, statistical method, other additional measurements)

### Result presentation

The extracted data will be presented in the form of graphs or tables in a manner that aligns with the objective and scope of this scoping review. A qualitative thematic analysis will be undertaken to provide an overview of the literature. We will summarize the commonness among similar experimental designs by the inductive method and explore the most appropriate task design for similar research objectives. Each table or chart will be accompanied by a narrative summary describing how the results relate to the review objectives and questions. The findings will be discussed based on practice and research, and a conclusion that is helpful to future studies in this field will be drawn.

## Discussion

Some scholars hold that the fMRI scan processes collecting data under the state of silence are supposed to be classified as the rest-state fMRI, such as the above-mentioned NRER design. Besides, the methods for data processing are ordinarily applied in the processing of rs-fMRI. In the traditional sense, task-based fMRI only includes block-design or event-related design paradigms. However, in terms of its implementation purpose, tb-fMRI studies are employed to explore the instant change of brain activities induced by some stimulations. In recent years, the underlying mechanism of acupuncture effects related to CNS is gradually revealed and lucubrated. Under such a background, researchers of acupuncture are also constantly refining and designing new approaches to investigate its instant effects and sustained effects.

From this point of view, these studies can be regarded as tb-fMRI instead of being classified only according to traditional views. There are still some conceptual problems because of a lack of summative studies or intensive discussions in this field. The research in this field is carried out through the imitation of each other without continuity.

Due to a large number of literature and different research methods, this review will adjust the data items to provide a better expression of information in the data extraction and result presentation. The objectives and designs of these studies will be summarized to make a critical evaluation or to propose quality requirements for future studies. Based on clinical practice or experimental practice, directional suggestions for further studies will be provided.

## Data Availability

All data generated or analyzed during this study will be included in the published scoping review article and will be available upon request.

## Abbreviations

TCM: Traditional Chinese medicine
CNS: Central nervous system
fMRI: Functional magnetic resonance imaging
PET: Positron emission tomography
EEG: Electroencephalography
MEG: Magnetoencephalography
BOLD: Blood-oxygen-level-dependent
rs-fMRI: Resting-state fMRI
tb-fMRI: Task-based fMRI
ReHo: Regional homogeneity
ALFF: Amplitude of low-frequency fluctuation
FC: Functional connectivity
ICA: Independent component analysis
NRER: Not-repeated event-related
CNKI: Chinese National Knowledge Infrastructure
CBM: Chinese Biomedical Medicine disc
